# Drug use in the management of uncomplicated malaria in public health facilities in the Democratic Republic of the Congo

**DOI:** 10.1186/s12936-018-2332-3

**Published:** 2018-05-03

**Authors:** Nsengi Y. Ntamabyaliro, Christian Burri, Didier B. Nzolo, Aline B. Engo, Yves N. Lula, Samuel M. Mampunza, Célestin N. Nsibu, Gauthier K. Mesia, Jean-Marie N. Kayembe, Joris L. Likwela, Leon M. Kintaudi, Gaston L. Tona

**Affiliations:** 1 Unité de Pharmacologie Clinique, Faculté de Medicine, Université de Kinshasa, Kinshasa, Democratic Republic of the Congo; 2 Division of Medicines Research, Swiss Tropical and Public Health Institute, Basel, Switzerland; 3 Department of Pharmaceutical Sciences, University of Basel, Basel, Switzerland; 4 Centre Neuropsychopathologique, Faculté de Médecine, Université de Kinshasa, Kinshasa, Democratic Republic of the Congo; 5 Département de Pédiatrie, Faculté de Médecine, Université de Kinshasa, Kinshasa, Democratic Republic of the Congo; 6 Département de Médecine Interne, Faculté de Médecine, Université de Kinshasa, Kinshasa, Democratic Republic of the Congo; 7 Programme National de Lutte Contre le Paludisme, Ministère de la Santé, RDC, Kinshasa, Democratic Republic of the Congo; 8 Santé Rurale, Kinshasa, Democratic Republic of the Congo; 9 Epidemiology for Global Health Institute, University of Antwerp, Antwerp, Belgium

**Keywords:** Uncomplicated malaria, Drug use, ACT, Quinine, Anti-malarials, Democratic Republic of Congo

## Abstract

**Background:**

Malaria the first causes of death from parasitic infection worldwide. Interventions to reduce the burden of malaria have produced a tremendous drop in malaria morbidity and mortality. However, progress is slower in DRC, which shares with Nigeria 39% of deaths related to malaria globally. Inappropriate use of drugs may be one of the factors of this below-average performance. The aim of this study was to describe the use of drugs in the management of uncomplicated malaria in public health facilities in DRC.

**Methods:**

A drug use study was carried out in DRC from January to March 2014. In each of the former 11 provinces of DRC, one Rural Health Centre, one Urban Health Centre and one General Hospital were selected. In each of them, 100 patient’s files containing prescription of anti-malarials from January to December 2013 were randomly selected. Among them, all of the files with diagnosis of uncomplicated malaria were included in this study. Prescribed anti-malarials, co-prescribed drugs and their indications were collected. Descriptive analyses were performed.

**Results:**

A total of 2300 files out of 3300 (69.7%) concerned uncomplicated malaria and were included in analysis. Malaria treatment was initiated after a positive RDT or microscopy in 51.5% of cases, upon suspicion without requesting biological confirmation in 37% and despite negative results in 11%. Twenty-nine (29) different treatment regimens were used. The drugs recommended by the National Malaria Control Programme were used in 54.3% of cases (artesunate–amodiaquine 37.4% or artemether–lumefantrine 16.9%). The second most used anti-malarial was quinine (32.4%). Apart from anti-malarials, an average of 3.1 drugs per patient were prescribed, among which antibiotics (67.9%), analgesics and non-steroidal anti-inflammatory (NSAIDs) (all abbreviations to be explicated on first use) (70.6%), vitamins (29.1%), anaemia drugs, including blood transfusion (9.1%) and corticosteroids (5.7%), In 51.4% of cases there was no indication for the concomitant medication.

**Conclusion:**

Management of uncomplicated malaria in DRC is characterized by a low adherence to treatment policy, numerous treatment regimens, and abundant concomitant medication potentially harmful to the patient. This may contribute to the low performance of DRC in malaria control. Determinant of this irrational use of drugs need to be assessed in order to formulate and implement efficient corrective measures.

## Background

Malaria is the first cause of death from parasitic infection worldwide. Developing tropical countries are the most affected. There were about 212 million cases of malaria in 2015 and an estimated 429,000 deaths among which 90% lived in the African region [1]. In the Democratic Republic of the Congo (DRC), 97% of the population is at risk of endemic malaria and the remaining 3% is exposed to malaria epidemics.

Fighting malaria involves many organizations at international and national level and the disease was included in the Millennium Development Goal (MDG 6c); which was to halt and begin to reverse the incidence of malaria by 2015 [2]. In 2005, the World Health Assembly (WHA) set the targets of reducing malaria case incidence rates by 75% by 2015 [2, 3]. As a result, the average malaria infection prevalence declined by 48% in children aged 2–10 years, mortality decreased by 47% worldwide and by 54% in the World Health Organization (WHO) Africa Region between 2000 and 2015, and 55 countries met the target set by the MDGs [1]. However, DRC is not on track to achieve the MDG or WHA targets; instead, it is among the 13 countries that carry 78% of the burden of malaria and in which the reduction of malaria incidence of 32% since 2000 is below the average of 53% in the other countries globally [2]. With about 1% of the world population [3] and 1.7% of those exposed to malaria, DRC had 9% of malaria cases and 10% of malaria deaths in 2015 and together with Nigeria a share of 36% of deaths related to malaria globally [1]. Inappropriate use of drugs may be among the determinants of this counter performance.

Inappropriate use of anti-malarials [4, 6], poor adherence to treatment guidelines [5, 7], lack of laboratory data supporting diagnosis of malaria before treatment [6, 8] or usage of drugs known to be ineffective [7] have been reported from African countries affected with malaria. In addition to anti-malarials, other drugs e.g., antibiotics, are irrationally used during malaria treatment [8, 10]. In DRC, an unpublished study of Mesia et al. found that the recommended ACT was only used by 6% of healthcare providers three years after implementation of artesunate/amodiaquine as first-line treatment for uncomplicated malaria. The situation had not changed much in 2014: 6% of children under 5 years with fever received an ACT [9].

There is an apparent need to improve rational use of anti-malarials, but without a knowledge basis of how drugs are being prescribed and used, it is difficult to initiate a discussion on rational drug use or to suggest measures to improve prescribing habits [10]. Taking this into account, it is of paramount importance to first assess the current practices in the use of anti-malarial drugs. The aim of this study is to describe the prescription of drugs in the management of uncomplicated malaria in Public Health facilities in DRC.

## Methods

### Study setting

DRC is the second largest and the fourth most populated country of Africa with a population estimated to 81 million inhabitants [11] The health system of DRC is composed of 515 Health Zones, each of them comprising one General Referral Hospital (GRH) and several Health Centres (HC). Most of them are integrated in the national health system and thus connected to the National Malaria Control Programme. Current, treatment policy of malaria in DRC [12] recommends that uncomplicated malaria is to be managed in HC, while severe malaria is managed in Referral Hospitals. HC are rural or urban according to their geographical location: rural areas are characterized by more poverty, less qualified health personnel, difficulties in transportation and all social services. Urban areas are characterized by more qualified health personnel, some better social services, but also more promiscuity. HC are mostly run by nurses especially in rural areas; physicians may sometimes be found in urban health centres but are mostly responsible of patient care in GRH. The study was carried out in all the previous 11 provinces of DRC. In each one of them, one GRH, one rural HC and one urban HC were selected to capture use of drugs in complicated, in uncomplicated malaria, as well as urban and rural conditions. DRC has been through many civil wars and there are still armed groups in many remote areas especially in the eastern regions. Therefore, the study was conducted in the Capital cities and the surrounding rural areas in each province. This is also where the population fleeing the abuses of the armed groups converge. The total catchment area covered by these health facility is approximately 4.5 million inhabitants according to the date provided by the Health Zones. The recommended drugs for uncomplicated malaria are artesunate + amodiaquine (ASAQ) or artemether + lumefantrine (AL).

### Study design

This was a cross-sectional drug use study that aimed at assessing the prescription of anti-malarial drugs. Therefore, a retrospective review of patients’ file containing a prescription of an anti-malarial from January to December 2013 was performed. One hundred files were randomly selected in each health facility as recommended by WHO in the investigation about drug use in health facilities [13], yielding a sample size of 3300 files. The data were collected from January to March 2014. Patients’ files were thereafter classified according to the diagnosis of malaria recorded by the prescriber: uncomplicated malaria, treatment failure, severe malaria, and are kept electronically as Excel spreadsheets.

Only the patients’ file in which the indication of anti-malarial prescription was uncomplicated malaria were included in this study. All cases of severe malaria or no malaria diagnosis were excluded from the analysis. For each of them, the following variables were recorded: Province, health facility (GRH, urban HC or Rural HC), age, and medical history; clinical signs and symptoms as well as laboratory results supporting the diagnosis of malaria; and the drug prescribed (anti-malarials and co-prescribed medication with their indication). Data were entered using Epi-info 7, CDC Atlanta, then exported in Microsoft Excel 2013. Descriptive analyses were performed. Calculation of 95% confidence interval was performed using Stata 12.

### Statistical analysis

Descriptive analyses were performed. Calculation of 95% confidence interval was performed using Stata 12.0 (StataCorp. 2011. Stata Statistical Software: Release 12. College Station, TX: StataCorp).

Variables were described by count or frequencies. When necessary, Chi square tests was used to compare them in children less than 5 years old with other patients. A p value < 0.05% was considered for statistical significance.

## Results

This drug use study was conducted in the 11 former provinces of DRC and analysed the patterns of the prescription of anti-malarial drugs in Health Centres and General Referral Hospitals. Of the 3300 patients’ files collected 2300 had uncomplicated malaria as the indication for anti-malarial drug prescription Tables 1 and 2.

**Table 1:** Age group (n = 2227)

Age group (year)	Number (%)
< 5	845 (37.9)
5–13	401 (18.0)
14–18	156 (7.0)
19–64	785 (35.3)
≥ 65	40 (1.8)

**Table 2:** Biological confirmation of malaria

Result of biological examination	Number	%	95% CI
Positive microscopy or RDT	1184	51.5	49.4–53.5
Negative microscopy or RDT	252	11.0	9.7–12.3
None	853	37.0	35.1–39.1
Positive microscopy, negative RDT	11	0.5	0.2–0.9
Total	2300	100	

### Patients’ characteristics

About one-third of the patients (37.9% of cases) are children under 5 years of age. Among them, 6.3% (140/2227) were less than 6 months old.

### Medical management

#### Confirmation of diagnosis prior to initiation of treatment

Confirmation of malaria by rapid diagnostic test (RDT) or microscopy was requested by the medical personnel only in 63% (1436/2300) of suspected cases; in 52% (1184/2300), the results were positive and in 11% (252/2300) of cases, malaria treatment was initiated despite negative results. In 2.6% of cases (59/2300), malaria diagnosis was based on both microscopy and RDT. Both were positive in 48 patients; in 11 cases, microscopy was positive but RDT negative. Table 3 shows that prescription of anti-malarial was based more frequently on positive results in children blow 5 years old.

**Table 3:** Biological confirmation in children under 5 years old compared to other patients

Result of biological examination	< 5 years, number (%) N = 845	≥ 5 years, number (%) N = 1455	p value
Positive microscopy or RDT	464 (54.9)	720 (49.5)	0.012058
Negative microscopy or RDT	79 (9.3)	173 (11.9)	0.059995
None	296 (35.0)	557 (38.2)	0.119552
Negative RDT positive microscopy	6 (0.7)	5 (0.3)	

#### Medical management of uncomplicated malaria

A total of 29 different treatments were used to manage uncomplicated malaria. The most used drugs were ASAQ, AL and quinine. As shown in Table 4 they were prescribed to 1994 patients (86.7%). They are are followed by the injectable artemisinin derivatives, alpha–beta arteether and artemether, then sulfadoxine-pyrimethamine (SP). The other drugs represented together 3.7%; each of them was used in less than 1% of patients. They include quinine containing treatments (quinine plus ACT, such as ASAQ, AL, dihydroartemisinin–piperaquine (DHA–PQ), quinine plus doxycycline, SP, artemether or artesunate); other ACT medicines, such as DHA–PQ and artesunate-SP and oral monotherapies, including oral artemisinin-based monotherapy (oAMT). Quinine and ASAQ were significantly more used in the subgroup of under 5 years old children the most affected by malaria whereas, artemether–lumefantrine and the injectable artemisinin derivative alpha–beta arteether were more used in the patients aged 5 years old and above.

**Table 4:** Treatment regimen for uncomplicated malaria

Anti-malarials	Number (%)	95% CI
ASAQ	860 (37.4)	35.4–39.4
QUININE	745 (32.4)	30.5–34.3
AL	388 (16.9)	15.4–18.5
A-β arteether	91 (4.0)	3.2–4.8
ARTEMETHER inj	56 (2.4)	1.8–3.2
SP	52 (2.3)	1.7–3.0
Quinine + ASAQ	23 (1.0)	0.6–1.5
Other	85 (3.7)	3.0–4.5
Total	2300 (100)	

#### Concomitant medication

Fifty-six patients (2.4%) received no other drug than anti-malarials. The other patient received an average of 3.1 drugs. Table 5 shows, for every concomitant medication, the number of patients that received it. Analgesics and NSAIDs were the most prescribed, to 70.6% of patients; antibiotics were prescribed to 68% of patients. Vitamins were prescribed to 29% of patients and the other anti-infectious drugs, mostly anti-helminthics were prescribed in 27.5% of cases. Antibiotics were used without a specified diagnosis in 56.2% of cases and for a non-bacterial infectious condition (vomiting, intestinal parasitosis, flu) in 16.6% of cases. Among antalgics, metamizole, a drug with known serious haematologic adverse reactions was prescribed to 12.2% of patients. Drug combinations used in influenza were prescribed to 6.1% of patients.

**Table 5:** Drugs concomitantly prescribed with anti-malarials

Drug groups (N = 2300)	Number of patients (%) [95% CI]	Most frequently co-prescribed drugs in the group (n; %)
Analgesics and NSAIDs	1624 (70.6) [68.7–72.5]	Paracetamol (1217; 52.9%), Metamizole (282; 12.3%), Aspirin (126; 5.5%), Diclofenac (80; 3.5%), Paracetamol/Ibuprofen (50; 2.2%), Ibuprofen (32; 1.4%), Tramadol (10; 0.4%)
Antibiotics	1561 (67.9) [66.0–69.8]	Amoxicillin (416; 18.1%), Metronidazole (303; 13.2%), Cotrimoxazole (266; 11.6%), Gentamicin (197; 8.6%), Ampicillin (171; 7.4%), Ciprofloxacin (158; 6.9%), Erythromycin (148; 6.4%)
Vitamins	670 (29.1) [27.3–31.0]	Multivitamins (348; 15.1%), vitamin C (124; 5.4%), Vitamin B1/B6/B12 (123; 5.3%), Vitamin B complex (76; 3.3%), vitamin B6 (48; 2.1)
Other anti-infectious drugs	633 (27.5) [25.7–29.3]	Mebendazole (524; 22.8%), Albendazole (50; 2.2%), Nystatin (29; 1.3%), Pyrantel pamoate (15; 0.7%), Clotrimazole (12; 0.5%), Levamisole (9; 0.4%)
Anaemia drugs	210 (9.1) [8.0–10.3]	Folic acid (68; 3.0%), Nutrisang (34; 1.5%), Hifer (31; 1.3%), VIT B12 (17; 0.7%), vitro z (13; 0.6%) Blood transfusion (11; 0.5%)
Rehydration solutions	195 (8.5) [7.3–9.6]	ORS (91; 4.0%), GLUCOSE SOLUTION (72; 3.1%), Ringer lactate (24; 1.0%), NaCl Solution (15; 0.7%)
Flu drugs	140 (6.1) [5.1–7.1]	Xylometazoline (74; 3.2%), Temperine (43; 1.9%), Rhinathiol (10; 0.4%); cold gripp (9; 0.4%)
Corticosteroids	130 (5.7) [4.7–6.6]	Dexamethasone (101; 4.4%), Prednisolone (17; 0.7%), Hydrocortisone (16; 0.7%)
Antiallergics	75 (3.3) [2.5–4.0]	Promethazine (52; 2.3%), Chlorpheniramine (17; 0.7%), Cetirizine (3; 0.1)
Antispasmodics	70 (3.0) [2.3–3.7]	Papaverine (43; 1.9%), Butyl scopolamine (27; 1.2%)
Antiemetics	51 (2.2) [1.6–2.8]	Metoclopramide (28; 1.2%), Ondansetron (22; 1.0%), Domperidone (1; 0.0%)

All age groups received concomitant medication. Table 6 shows that, significantly more children under five were prescribed antibiotics compared to other patients (75% vs. 63%). Antalgics, (74.7% vs. 68.3%), drugs used for anaemia, among which, iron salts, folate and vitamin B12 and blood transfusion (16.2% vs. 4.9%). Ten of the 11 blood transfusions were given to children of this age group. For the one remaining transfusion, age was not specified but the weight of the patient (8.5 kg) strongly suggests he is in this same age group. Corticosteroids and drugs used in the management of influenza were also significantly more prescribed to children under five. Prescription of vitamins was comparable in both groups.

**Table 6:** Concomitant medication in children under 5 years old compared to other patients

Drugs	< 5 years, number (%) N = 845	≥ 5 years, number (%) N = 1382	p value
Antibiotics	634 (75.0)	876 (63.4)	< 0.00001
Antalgics and NSAIDs	631 (74.7)	944 (68.3)	0.001353
Vitamins	245 (29.0)	402 (29.1)	0.962106
Other anti infectious	254 (30.1)	362 (26.2)	0.047849
Anemia drugs	137 (16.2)	68 (4.9)	< 0.00001
Rehydration	83 (9.8)	97 (7.0)	0.0185
Flu drugs	74 (8.8)	61 (4.4)	0.000031
Corticosteroids	67 (7.9)	57 (4.1%)	0.000145

Prescription of antibiotics was found to be comparable (p = 0.137802) in patients with positive biological confirmation, of malaria 69.1% (818/1184) and those with negative tests 74.2 (186/252). Surprisingly however, antibiotics were significantly less prescribed (p = 0.025078) in patients with no biological confirmation of malaria, 64.4% (549/853) than in patients with positive biological confirmation.

## Discussion

Despite the use of the same powerful dugs, DRC is still heavily affected by malaria and concentrates 10% of all deaths related to malaria worldwide, most of whom are children under 5 years old. Inappropriate use of drugs in the management of this disease may play a significant role in this counter performance. This study aimed at assessing the use of drugs in the management of uncomplicated malaria in rural and urban health zones and referral hospitals. In each of the 11 provinces of DRC, only those in or near the capital cities were included. This is also where converge the population fleeing the abuses of armed groups. The stratification by province, and health facility, as well as the consideration of rural and urban aspects may contribute to increase the representativeness of our sample. In addition, the preponderance of patient aged less than 5 year could mean a rash between our sample and the population affected by malaria in DRC [9]. It is worth to notice the 6.3% of infants under 6 months. Malaria was thought to be rare in this age group for various reasons [14]. But more and more data suggest that malaria may not be as rare as previously thought and may be masquerading other severe infectious conditions [14] Thus; there is a need to understand more about the characteristic of malaria in this age group as well as their optimal treatment. The WHO has recognized the infants under 12 months [16] as one of the most vulnerable population. Use of long-lasting insecticidal nets (LLINs), intermittent preventive therapy with SP for infants (IPTi) and prompt diagnosis and effective treatment of malaria infections [16] have been recommended. For some of these infants, especially those weighing less than 5 kg, there is a lack of proven treatment options: none of the current ACT is approved for infants under 4.5 kg, and there are concerns about the safety of quinine in this age group [17] and, as shown for AL [18], pharmacokinetics of some drugs recommended for other age group, can be different in this group, leading to increased exposure to these substances. There is still a need for evidence to understand more about the best treatment for this vulnerable population that represents a non-negligible proportion of the malaria patients.

Malaria treatment was initiated without biological confirmation in almost half of the cases. This may results in wastage of drugs that should be given to true malaria cases [19], unnecessary and avoidable adverse drug reactions (ADR) and delay in the detection of the true illness of the patient which can be lethal [20]. Indeed the WHO and the Congolese NMCP have recommended biological confirmation of malaria by either RDT or microscopy before initiation of treatment. However, the performance of diagnostic tests prior to treatment is not a guarantee of optimal use of anti-malarials. Malaria treatment initiated despite negative results in 11% of cases which is in line with other studies [21, 22] with a rate of malaria treatment despite negative laboratory testing diagnosis of as high as 58% in Nigeria [21]. This inappropriate prescription of anti-malarials despite negative results may be caused by the assumption of undetectable malaria, lack of tools for alternative diagnosis, provider know-how and time, the patients demand for drugs [23], lack of trust in laboratory results, constancy of anti-malarial supply and resistance to change from pre-existing patterns [24]. Another issue is the drug prescribed: The recommended ACT formulations, ASAQ and AL, were prescribed in only half of the cases. Patients below 5 years old were more likely to be prescribed ASAQ or quinine whereas the older patient received more AL and alpha–beta arteether. Currently, ACT is the best choice for management of uncomplicated malaria. The demonstrated efficacy and safety of ASAQ [25, 26] and AL [27, 28] have led the NMCP of DRC to recommend them [12] for management of uncomplicated malaria. On the other hand quinine use in malaria treatment was established before the methods for modern clinical trials were developed [29]; this drug has a low therapeutic index [17], which means an increased risk of dose-related adverse reactions. In addition, head to head studies have shown superiority of ACT over quinine in the management of uncomplicated malaria [30, 31]. Despite these evidences, quinine continue to be used (up to 40% in children under 5 years old). One of the reasons would be that this drug has been used against malaria for a long time. The drug is so popular that in many areas of DRC that the word quinine has been included in local languages as a word for any tablet. Yet, the widespread usage of quinine may probably be playing a role in the low performance of DRC in malaria mortality and morbidity. The injectable artemether and alpha beta arteether were also used despite they are not recommended for uncomplicated malaria and there is no WHO prequalified product available [32]. The use of these injectable monotherapies in uncomplicated malaria may also fuel drug resistance like the oral artemisinin-based monotherapies (oAMT). Oral monotherapy of artesunate, dihydroartemisinin (DHA) and amodiaquine were used in 3, 4 and 1 patients, respectively. This seems very low, but there is still a concern: the WHO has identified use of oAMT as one of the major driver of resistance to artemisinin and its derivatives [33]. The regulatory authority of DRC has responded by withdrawing marketing authorization to oAMT [34], but these drugs are still prescribed and this raises also the question of the origin of these oAMT, their presence in pharmacies and unauthorized drug sellers and why they are still prescribed by health professionals. Compliance to treatment guidelines is a major factor of treatment success [35]. However, prescribers adhered to treatment policy in only half of the cases which is in line with findings from other countries in Sub-Saharan Africa [6–8]. This may be related to many factors like stock-outs, ignorance, the attempt to save money, difficulties to change habits or misleading incentives. Better organisation of health facilities and training of personnel may significantly increase adherence to national policy as shown in Uganda [36]. Nevertheless, adherence to treatment guidelines by health workers is not the end of the story; it is important to take in account adherence of the patient to the prescription. Onyango et al. have shown that adherence to prescription was very low especially in the poorest, less educated and younger patients [37]. So when assessing factors related to low adherence of prescribers to treatment policy, it is important to take in account the adherence of patients to treatment and related factors in order to put in place more adapted corrective measures. In addition to anti-malarials, the patients received other concomitant medication.

Uncomplicated malaria presents in the form of numerous unspecific signs and symptoms including most of the time headaches, fever, and pain which drive the prescription of antipyretic drugs, analgesics or NSAIDs. Paracetamol was the most prescribed, however, metamizole a drug known to induce rare but irreversible agranulocytosis [38, 39] was given to 12.3% of patients. Given this risk and considering the multiple alternatives the use of metamizole seems inappropriate.

More than 2/3 of patients were prescribed antibiotics. Children blow 5 years old were significantly more likely to be prescribed antibiotics, as shown in other studies [8, 40]. The lack of confirmation of malaria or negative results are among the drivers of antibiotic co-prescription with anti-malarials [41] But, surprisingly, prescription of antibiotics was comparable in patient with positive and negative parasitological exams. Patient for which confirmation of malaria was not sought were prescribed rather less antibiotics. Some Congolese prescribers assume inexplicably that better management of malaria requires addition of antibiotics to anti-malarials. Whether this is a misunderstanding of the recommendation to combine quinine with clindamycin or doxycycline, or no is not known. Nevertheless, this remains an issue that needs an assessment of extent and determinants in Congolese settings because it may be part of the irrational use of antibiotics which is common especially in developing countries and a major contributor to bacterial resistance [8, 42]. Considering the high prevalence of malaria in DRC and the high prescription of antibiotics for malaria, management of malaria may be threatening treatment of bacterial infection and this can cause significant problems to patients and health systems.

Vitamins, mostly multivitamins were prescribed to 29.1% of patients, without an indication mentioned in the files. There was no significant difference in prescription to children under-five compared to other patients. Neither the WHO guidelines nor the national guidelines recommend usage of vitamins in the management of malaria. Some studies have speculated that the antioxidant effect of vitamins, especially vitamin C and E, may reduce efficacy of artemisinin derivatives [43]. The same concern may be evoked regarding the use of iron whose supplementation in children has been shown to increase susceptibility to malaria [44, 45]. The effect of iron supplementation on the efficacy of ACT has not been assessed; however, a study in Malawi [46] showed reduction in efficacy of sulfadoxine-pyrimethamine in children receiving concomitantly SP and iron supplementation. Other studies [47, 48] has shown no beneficial effect iron supplementation during malaria treatment. In DRC where, 60% of children aged 6–59 months are affected by anaemia [9], these interactions need to be more assessed in order to propose more efficient strategies.

Blood transfusion in the context of management of malaria is used to improve oxygen delivery to tissue, due to severe anaemia. Eleven children under five received blood transfusion and the recorded indication was just anaemia. In severe malaria, blood transfusion may be necessary, but benefits of this procedure need to be weighed against risks of transmission of serious infectious diseases (HIV, hepatitis B) severe immunologic reactions, volume overload, hyperkalaemia [49]. Transfusions are necessary when the level of haemoglobin is so low that it is not tolerated and that may define severe malaria [29]. These 11 transfusions may be related to misclassification of complicated malaria cases as uncomplicated, or to the numerous unnecessary transfusions given to ambulatory patients.

## Conclusion

DRC is still heavily affected by malaria despite the effectiveness of available tools to control this potentially lethal disease. Inappropriate use of those tools, namely medicines still hampers better control of the disease. Half of the patients receive malaria treatment with no diagnostic confirmation and adherence to treatment guidelines is low. Quinine, which is no more recommended for uncomplicated malaria is still widely used. In addition, the management of uncomplicated malaria is characterized by polypharmacy, mostly in children below 5 years old. Dangerous analgesics like metamisole are used. Antibiotics and other drugs, like vitamins, anaemia drugs and corticosteroids are prescribed, most of the time without an indication. The massive use of antibiotics raises the concerning question that management of uncomplicated malaria in DRC may be one of the drivers of selection of resistant bacteria which is a serious threat to public health. All these practices may be threatening the success of the control of malaria in DRC. There is a need to train all stakeholders in the proper management of uncomplicated malaria and make available all diagnostic tools, especially RDTs and the recommended drugs. The withdrawal of the non-recommended drug from the marked will also improve the management of uncomplicated malaria. The determinants of all these situations need to be assessed in order to formulate more accurate recommendations.

## Abbreviations

ACT: Artemisinin-based combination therapy
ADR: adverse drug reaction
AL: artemether plus lumefantrine
ASAQ: artesunate plus amodiaquine
DHA: dihydroartemisinin
DRC: Democratic Republic of the Congo
GRH: General Reference Hospital
HC: Health Centre
IV: intravenous
MDG: millennium development goals
NMCP: National Malaria Control Programme
NSAID: non-steroidal anti inflammatory
oAMT: oral artemisinin based monotherapy
PQ: piperaquine
RTD: rapid diagnostic test
SDG: sustained development goals
WHO: World Health Organisation
